# Distinct placental malaria pathology caused by different *Plasmodium berghei* lines that fail to induce cerebral malaria in the C57BL/6 mouse

**DOI:** 10.1186/1475-2875-11-231

**Published:** 2012-07-16

**Authors:** Lurdes Rodrigues-Duarte, Luciana Vieira de Moraes, Renato Barboza, Claudio RF Marinho, Blandine Franke-Fayard, Chris J Janse, Carlos Penha-Gonçalves

**Affiliations:** 1 Instituto Gulbenkian de Ciência, Rua da Quinta Grande, 06, Oeiras, 2780-156, Portugal; 2 Department of Parasitology, Instituto de Ciências Biomédicas, University of São Paulo, São Paulo, Brazil; 3 Leiden Malaria Research Group, Parasitology, Leiden University Medical Center, Leiden, The Netherlands

**Keywords:** *Plasmodium berghei*, Placental malaria, Cerebral malaria, Placental pathology, TNF, TLR4, TLR2

## Abstract

**Background:**

Placental malaria (PM) is one major feature of malaria during pregnancy. A murine model of experimental PM using BALB/c mice infected with *Plasmodium berghei* ANKA was recently established, but there is need for additional PM models with different parasite/host combinations that allow to interrogate the involvement of specific host genetic factors in the placental inflammatory response to *Plasmodium* infection.

**Methods:**

A mid-term infection protocol was used to test PM induction by three *P. berghei* parasite lines, derived from the K173, NK65 and ANKA strains of *P. berghei* that fail to induce experimental cerebral malaria (ECM) in the susceptible C57BL/6 mice. Parasitaemia course, pregnancy outcome and placenta pathology induced by the three parasite lines were compared.

**Results:**

The three *P. berghei* lines were able to evoke severe PM pathology and poor pregnancy outcome features. The results indicate that parasite components required to induce PM are distinct from ECM. Nevertheless, infection with parasites of the ANKAΔ*pm4* line, which lack expression of plasmepsin 4, displayed milder disease phenotypes associated with a strong innate immune response as compared to infections with NK65 and K173 parasites.

**Conclusions:**

Infection of pregnant C57BL/6 females with K173, NK65 and ANKAΔ*pm4 P. berghei* parasites provide experimental systems to identify host molecular components involved in PM pathogenesis mechanisms.

## Background

Organ pathology evoked by *Plasmodium* infections often correlates with accumulation of infected erythrocytes in specific organs leading to severe clinical manifestations as is the case of respiratory distress, cerebral malaria (CM) and severe placental malaria (PM) [1]. PM is one major feature of malaria during pregnancy and is usually associated with low birth weight due to intra-uterine growth retardation and/or preterm delivery ( [2] and reviewed in [3]), stillbirths, maternal anaemia and mortality [4,5]. Placental malaria results from accumulation of parasitized erythrocytes that is associated with a prominent monocytic inflammatory response that entails increased IFN-γ and TNF production and enhanced levels of monocyte/macrophage recruiting factors (MIP-1α and MIP-1β) [1,6]. Placental malaria pathology includes maternal-foetal barrier thickening, disorganization and destruction of placental tissue, proliferation of cytotrophoblastic cells and excessive perivillous fibrinoid deposits usually associated with focal syncytiotrophoblastic necrosis [7-10]. The severity of placental pathological manifestations is associated with a spectrum of severe pregnancy outcomes but the host cellular and molecular components that control the intensity of the inflammatory response are still not well-defined and are difficult to investigate in pregnant women.

An experimental system where *P. berghei* ANKA evokes a syndrome that resembles severe PM in women was established in a experimental cerebral malaria (ECM)-resistant mouse strain (BALB/c) allowing experimental investigation of PM pathogenesis in the mouse. Low foetal viability and increased maternal disease severity correlate with placenta pathology that, in this experimental model, is characterized by thickening of the placental barrier in the labyrinth zone and tissue damage, accumulation of monocyte/macrophages and enhanced expression of pro-inflammatory, apoptosis and oxidative stress factors [11-13]. The use of the *P. berghei* model of malaria for analysis of PM would benefit by development of additional experimental tools. Access to numerous (C57BL/6) mouse mutants would allow interrogating the involvement of host genetic factors in the placental inflammatory response to *Plasmodium* infection. Recently, the C57BL/6 mouse strain in combination with the rodent parasite *Plasmodium chabaudi* has been exploited to study pregnancy malaria pathogenesis with infection initiated early in gestation [14,15]. Here, different parasite lines derived from the *P. berghei* strains K173, NK65 and ANKA Δ*pm4*[16] that are not able to induce ECM in C57BL/6 mice, were used to establish additional placental malaria experimental models in this mouse strain. The results show that pregnant mice infected with the three lines develop PM indicating that *P. berghei* parasite factors that are responsible for inducing ECM in the C57BL/6 mouse are not required to induce placental pathology and poor pregnancy outcome in female mice infected during pregnancy. These experimental systems are valuable tools to study host and foetal genetic factors in the pathogenesis of placental response to *Plasmodium* infection.

## Methods

### Mice and pregnancy monitoring

Eight to twelve week-old C57BL/6 mice were obtained from the animal facility at Instituto Gulbenkian de Ciência. Mice were bred and maintained under specific-pathogen free (SPF) conditions. C57BL/6 females were transferred to a cage with one isogenic male (two females: one male) and removed after 48 hours. The day the females were removed was considered gestational day 1 (G1). Pregnancy was monitored every other day by weighing females. Successful fertilization was confirmed between G10 and G13 when animals had an average increase of 3 to 4 g in body weight. Abrupt weight loss after G13 was an indicator of unsuccessful pregnancy. Animal housing and all procedures were in accordance with national regulations on animal experimentation and welfare and approved by the Instituto Gulbenkian de Ciência Ethics Committee.

### Parasites and infection

**Figure 1:**
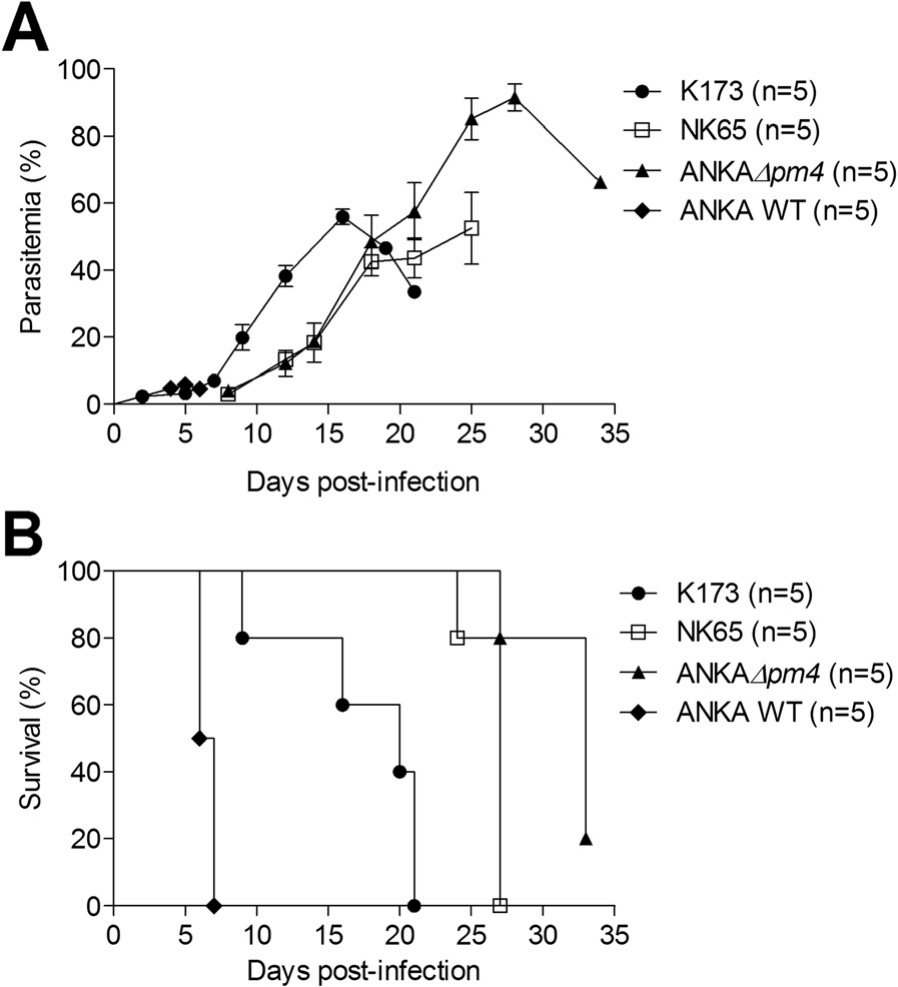
**Susceptibility to infection of C57BL/6 mice.** (**A**) Time-course parasitaemia and (**B**) survival of *P. berghei* K173, NK65 and ANKA*Δpm4* in C57BL/6 non-pregnant mice. Animals were infected i.p. with 10^6^ IE. Parasitaemia of DRAQ-5 labelled samples was followed by FACS. In (**B**) survival curves were compared using the Log-Rank (Mantel-Cox) test. Statistical significance results were *p*<0.01 when comparing K173 *vs* NK65 or ANKA*Δpm4*; *p*<0.05 when comparing NK65 to ANKA*Δpm4.*

The following parasite lines were used in this study: i) A reporter parasite line of the K173 strain/isolate of *P. berghei* which expresses the reporter protein GFP-luciferase under the control of the schizont-specific *ama-1* promoter*.* This mutant (line 1272cl1) has been generated in the K173cl1 line [14]. The *gfp-luciferase* gene has been integrated into the c/d-ssu-rRNA unit by double cross-over integration without a drug selectable marker. Details of this line can be found in the RMgmDB database [17]; ii) A mutant of *P. berghei* ANKA which lacks expression of plasmepsin-4 (ANKA*Δpm4;* line 1092cl4; RMgmDB-316) and expresses the reporter fusion protein GFP-luciferase under the control of the *ama-1* promoter [16]; iii) a parasite line originally derived from the *P. berghei* isolate NK65 at New York University and kindly provided by Dr Maria Mota (Instituto de Medicina Molecular, Lisbon, Portugal). Infections in Figure 1 were performed by intraperitonial (i.p.) injection of 10^6^ infected erythrocytes (IE). Parasitized red blood cell preparations were obtained from one *in vivo* passage in C57BL/6 mice, when the percentage of infection reached approximately 10%. Pregnant mice were intravenously (i.v.) injected with 10^6^ infected erythrocytes. Infection with ANKA*Δpm4* at G13 yielded very low parasite burden during pregnancy due to reduced multiplication rate of this parasite [16], but infection at G10 allowed significant parasite expansion within pregnancy time. Thus, infection was performed on G10 (ANKA*Δpm4* IE) or G13 (*P. berghei* K173 or NK65 IE). Parasitaemia was measured by flow cytometry [18] to detect infected erythrocytes stained with DRAQ5 (Biostatus Limited). The labelling of infected red blood cells with DRAQ5 is an adaptation of the manufacturer’s protocol for cell cycle analysis by flow cytometry. Briefly, a drop of blood was collected by tail pinching of infected mice into 400 μl of FACS Buffer (PBS 1x, 2% FBS, sodium azide 0.02%). DRAQ5 was added directly to the collected samples at a final concentration of 1μM. Samples were vortexed to allow an appropriate incorporation of DRAQ5 into the parasite DNA and immediately analysed. Uninfected red blood cells do not stain positive for DRAQ5 as they are devoid of DNA content. Parasitaemia was expressed as % of stained cells within the erythrocyte morphological gate. ECM development was monitored from day 5 post-infection (PI) as including one or more of the following neurological symptoms; head deviations, paralysis, ataxia and convulsions [19].

### Pregnancy outcome and foetal survival

Infected pregnant mice were killed by CO_2_ narcosis and subjected to caesarian section on G18 (K173 or NK65 infection) or G19 (ANKA*Δpm4* infection) and stillbirths, foetal weight, foetal survival at delivery and placental pathology were evaluated. Foetuses were extracted from their amniotic envelop and viability was immediately evaluated by reactive movement to touching with pliers. The lack of prompt movement indicated that the foetus had recently died. Reabsorptions were identified as small implants with no discernible foetus and placenta, corresponding to embryos that died before complete placenta vascularization. Viable and non-viable foetuses were weighed and counted. Non-viable (dead foetuses plus reabsorptions) foetuses were recorded as stillbirths. Viable foetuses were killed combining hypothermia and CO_2_ narcosis. In another set of experiments, infected pregnant mice were allowed to deliver in order to access litter size and newborns viability. Foetuses that have been expelled before the gestational day of analysis were also recorded as dead newborns. Non-infected pregnant mice were used as controls.

### Placenta preparations and morphometric analysis

Placentas from infected and non-infected females sacrificed on the same gestational day were equally treated. Each placenta was separated in two halves: one half was fixed in 10% formalin for further histological processing and the other half collected in lysis buffer (RNeasy Mini Kit - Qiagen) 1% β-mercaptoethanol for RNA extraction. Paraffin-embedded non-consecutive placenta sections were stained with hematoxylin-eosin (HE). HE-stained placental sections were analysed for histopathology and vascular space quantification. In each section, three randomly selected microscopic fields in the labyrinthine region (magnification 400x) were acquired at 12 Mpixels resolution, using a colour video camera (AxionCam HRc, Zeiss) connected to a light microscope (Axion Vision, Imager.M2, Zeiss). To quantify vascular spaces only, areas that presented accumulation of glycogen cells, necrosis or thrombi were excluded. An image analysis routine using ImageJ (ImageJ 1.37v, National Institutes of Health) was implemented. Briefly, after acquisition, the images underwent an automated light analysis procedure where noise removal was applied to ensure colour and image quality standardization across sections and specimens. Images were given a colour threshold to cover the area corresponding to blood spaces lumen. The coverage percentage was calculated as the ratio between the number of pixels covered by the area defined by the threshold and the overall number of pixels in the image. The blood vascular area in each placenta was estimated from the analysis of three non-consecutive sections. Two independent observers analysed the placentas one of which was unaware of the samples identification. Results were reported as the average of counts obtained by the two observers.

### RNA isolation and gene expression analysis

Total RNA from individual placentas was obtained using an RNeasy Mini Kit (Qiagen), following the manufacturer’s instructions for animal tissues. One microgram of total RNA was converted to cDNA (Transcriptor First Strand cDNA Synthesis Kit, Roche) using random hexamer primers. Ccl2, Ccl3, Tlr2, Tlr4 and TNF expression was quantified using TaqMan Gene Expression Assays from ABI (Mm00441242_m1, Mm00441258_m1, Mm00442346_m1, Mm00445273_m1, Mm00443258_m1, respectively). For *P. berghei* ANKA quantification specific primers for Taqman were, Forward 5’-CCG ATA ACG AAC GAG ATC TTA ACC T–3’, Reverse 5’- CGT CAA AAC CAA TCT CCC AAT AAA GG-3’ and Probe 5’– ACT CGC CGC TAA TTA G -3’ (FAM/MGB). The endogenous control Gapdh (Mouse GAPD Endogenous Control, ABI) was used in multiplex PCR with target genes. PCR reactions were performed with ABI Prism 7900HT system. For TaqMan assays, ΔCt was calculated by subtracting the cycle threshold (Ct) of the target gene from the GAPDH and relative quantification was obtained with normalization by GAPDH. Results were plotted as fold change over the respective non-infected controls.

### Statistical analysis

Survival curves were compared using the Log-Rank test (Mantel-Cox). Parasitaemia data were presented as mean values +/- SEM. Unpaired t test (WelchÂ´s correction) was performed in comparison of each parasite line with the respective control group. Kruskal-Wallis non-parametric test with Dunn’s post-test was used for comparisons between the three infected groups. Data were considered significant when *p*<0.05

## Results

### Increased parasitaemia in pregnant mice

**Figure 2:**
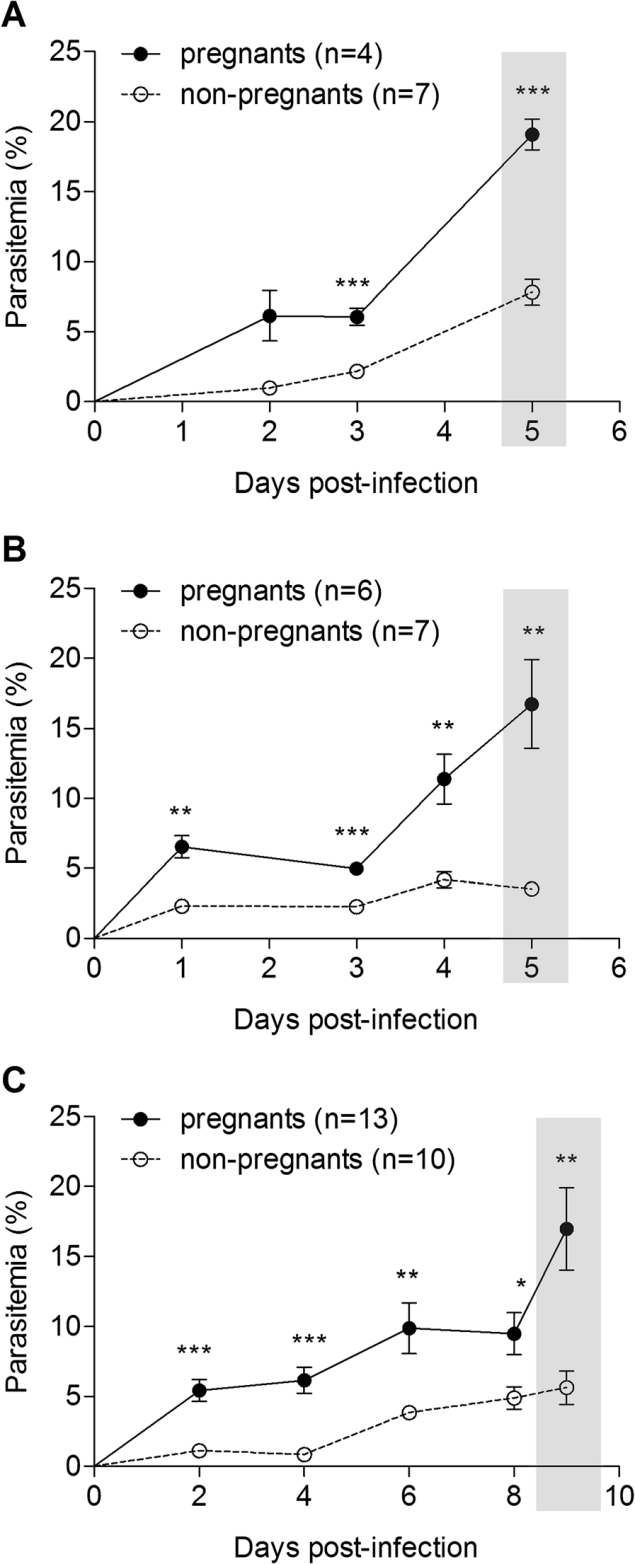
**Maternal susceptibility to infection.** (**A**) Time-course parasitaemia of *P. berghei* K173 (**A**), NK65 (**B**) and ANKA*Δpm4* (**C**) in C57BL/6 pregnant and non-pregnant females. Animals were infected i.v. with 10^6^ IE at G13 (A, B) or at G10 (C). Highlighted area corresponds to G18 (**A**, **B**) or G19 (**C**). Parasitaemia of DRAQ-5 labelled samples was followed by FACS. Unpaired t test (WelchÂ´s correction) *p<0.05; ** *p*<0.01; ***p<0.001.

As opposed to the canonical strain *P. berghei* ANKA, parasites of the NK65 isolate and of mutant ANKA*Δpm4* have been previously described not to cause experimental cerebral malaria (ECM) in the C57BL/6 susceptible strain [13,15]. Similarly, the K173cl1 line derived from the K173 isolate has lost the capacity to induce ECM [unpublished results, CJJ and BF]. The course of infection by the three parasite variants in non-pregnant C57BL/6 females (Figure 1) showed that differently from wild-type *P. berghei* ANKA the three parasite lines do not induce the characteristic features of ECM. Nevertheless they induced hyperparasitaemia leading to a fatal outcome. *P. berghei* ANKA*Δpm4* and NK65 exhibited slower growth kinetics when compared to the K173 line and corresponding delayed effects on the survival rate. To ascertain the effect of infection in pregnancy, mice were infected with K173 and NK65 at G13 when placenta vascularisation is established while infection with ANKA*Δpm4* was performed at G10 due to reduced multiplication rate of this parasite [16]. Pregnant mice did not exhibit symptoms of ECM but showed higher parasitaemia across time as compared to non-pregnant females, reaching 20% at 5 to 9 days after infection (Figure 2) and indicating that parasites of the three lines show increased ability to cause hyperparasitaemia during pregnancy.

### Impaired pregnancy outcome

**Figure 3:**
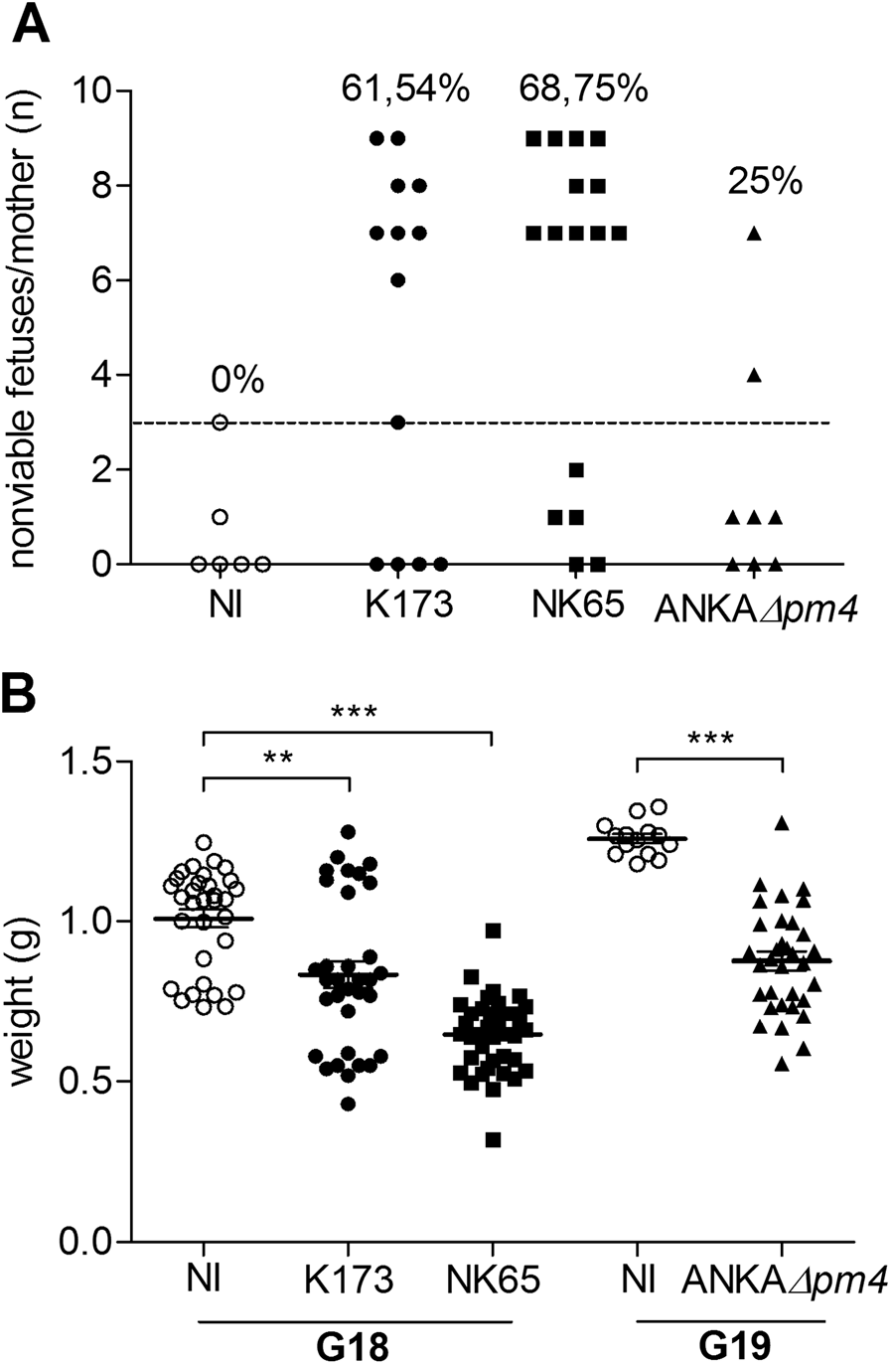
**Stillbirths and underweight foetuses as consequence of infection.** C57BL/6 pregnants were infected i.v. on G13 (K173 or NK65) or on G10 (ANKA*Δpm4*) with 10^6^ IE. Number of stillbirths (dead foetuses and/or reabsorptions) per mother *in utero* (**A**) and viable and non-viable foetuses weight (**B**) were accessed at G18 or G19. **p<0.01; ***p<0.001; NI: non-infected. Cut-off for non-viability was established in non-infected pregnant mice (dashed line).

The impact of infection in the pregnancy outcome was ascertained by the frequency of stillbirths *in utero* as well as foetal weight at G18 in pregnant females infected with NK65 and K173 or at G19 in ANKA*Δpm4* infected mice. Intra-uterine foetal death was increased in pregnancies of NK65-infected mice (69% of the females had a high number of non-viable foetuses), in K173-infected mice (62%) and in ANKA*Δpm4* infected pregnants (25%) when compared to non-infected controls (Figure 3A and Additional file 1). Viable and non-viable foetuses from NK65, ANKA*Δpm4* and, to a lesser extent, from K173-infected mothers showed significantly reduced weight as compared to non-infected controls at the same gestational day (Figure 3B). Moreover, newborn viability at delivery was strikingly reduced (Table 1) as compared to non-infected pregnant females. In some instances NK65 infection provoked maternal death during pregnancy (Table 1). These results show that infection of C57BL/6 pregnant females with *P. berghei*-derived parasite lines that fail to induce ECM, recapitulate with different degrees of severity the features of PM described in BALB/c mice infected *P. berghei* ANKA [11,12] namely, intrauterine growth retardation and poor pregnancy outcome. Nevertheless, it was clear that infection with ANKA*Δpm4* had milder effects on pregnancy outcome.

**Table 1:** Newborn reduced viability after maternal infection during pregnancy

	**NI**	**K173**	**NK65**	**ANKA*Δpm4***
Number of mothers	5	4	3*	4
Number of newborns	40	24	16	22
Nr. Dead newborns (%)	2 (0.5)	22 (91.6)	16 (100)	15 (68)

NI: non-infected; * Number of surviving mothers from a initial group of 5.

### Placental pathology in C57BL/6 mice

**Figure 4:**
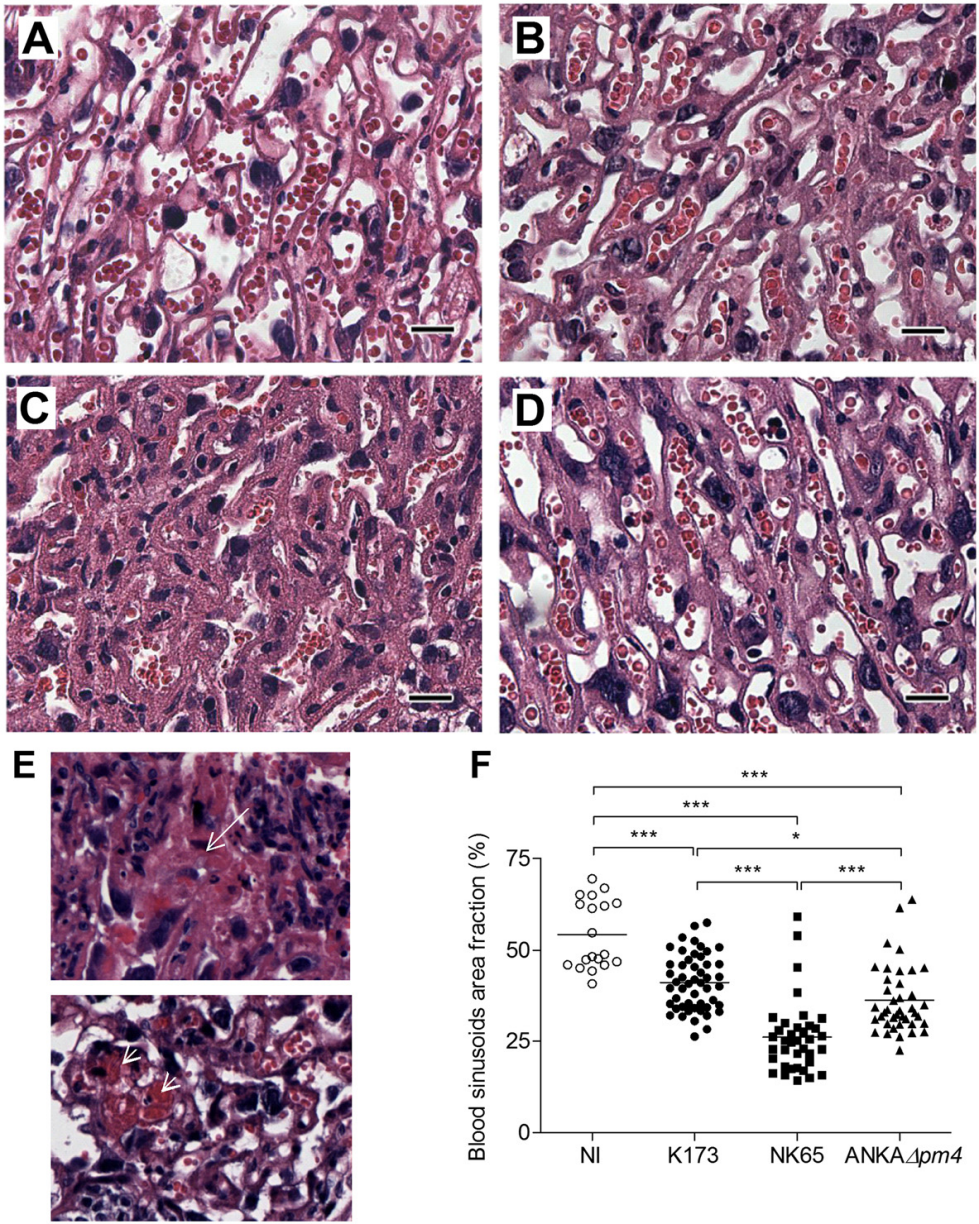
**Placental histology of labyrinth zone and morphometric analysis.** Representative photomicrograph of H&E stained placental sections of non-infected (NI) (**A**), K173 (**B**), NK65 (**C**) and ANKA*Δpm4* infected pregnant mice (**D**). (**E**) Histological sections show necrotic foci (arrow) and thrombi (arrowhead) in placentas infected with ANKA*Δpm4* (upper picture) and K173 (lower picture) parasites. (**F**) Relative quantification of vascular space using an automated morphometric procedure in H&E stained sections evaluated in placental sections. Mice were infected i.v. with 10^6^*P. berghei* K173 or NK65 IE (G13) or ANKA*Δpm4* IE (G10); placentas were excised on G18 (K173 and NK65) or G19 (ANKA*Δpm4*). Scale bar: 20 μm; in (**F**) line refers to mean; *p<0.05;**p<0.01; ***p<0.001.

**Figure 5:**
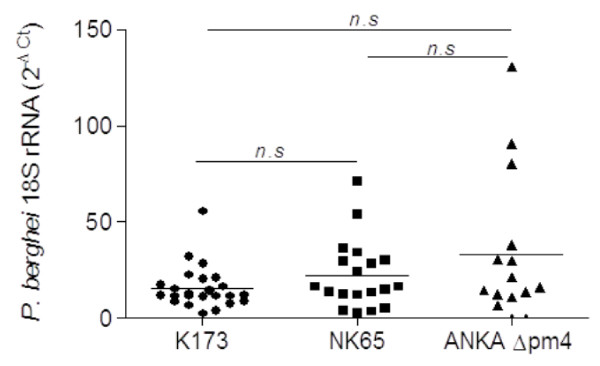
**Placental parasite burden.** Placentas were collected at G18 (K173 and NK65) or G19 (ANKA*Δpm4*) and RNA expression of *P. berghei* was evaluated by qReal Time PCR. ΔCT was calculated by subtracting the cycle threshold (Ct) of the target gene from the GAPDH. DunnÂ´s Multiple Comparison Test, *p*>0.05 (n.s.).

Next, infected placentas were examined for pathological features typical of PM including syncytiotrophoblast thickening, tissue destruction, fibrin deposits, thrombi formation and reduction of maternal blood space [11]. HE-stained sections displayed variable degrees of localized trophoblast layer thickening, placental tissue disorganization (Figure 4 B-D) and associated necrosis foci in the labyrinth zone and thrombi (Figure 4E) as compared to non-infected placentas (Figure 4A). Morphometric analysis of the labyrinth zone revealed a significant reduction in the available area for maternal blood circulation in all infected groups when compared to non-infected controls but NK65-infected mothers showed the highest restriction in blood space area (Figure 4F). Thus, the severity of placental pathological alterations evoked by the three parasite lines is distinct which corroborated the observed differences in pregnancy outcome. Notwithstanding the observed differences in pregnancy outcome and placenta pathology, the infection with these three parasite lines did not result in significant differences in placental parasite burden (Figure 5). These results indicate that despite the observed differences in placental pathology and pregnancy outcome, the ability to accumulate in the placenta is not significantly different between the three *P. berghei* lines.

### Differential patterns of placental inflammation

**Figure 6:**
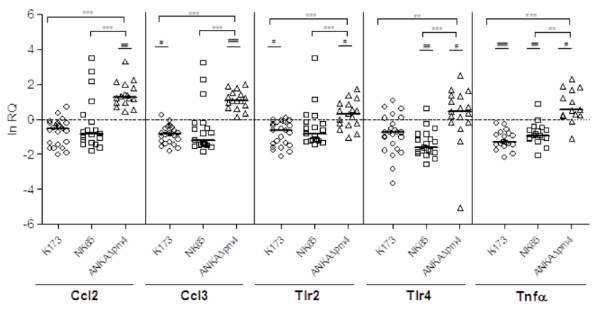
**Placental gene expression of inflammatory factors.** Placentas were collected at G18 (K173 and NK65) or G19 (ANKA*Δpm4*) and RNA expression of Ccl2, Ccl3, Tlr2, Tlr4 and Tnfα were evaluated by qReal Time PCR. Relative quantification (RQ) was obtained with normalization by GAPDH. Results are plotted as fold change against non-infected controls collected at the same gestational day (G18 versus K173 or NK65 and G19 versus ANKAΔpm4); Line refers to median values. Kruskal-Wallis Test **p<0.01; ***p<0.001 refers to differences between different parasite lines; Unpaired t test #*p*<0.05; ##*p*<0.01; ###*p*<0.001 compares each parasite line to its respective non-infected control.

To investigate the differences in pregnancy outcome and placental immune response to infection elicited by the three *P. berghei* lines, gene expression of pro-inflammatory molecules and informative markers of innate and adaptive immune responses in infected placentas relative to non-infected controls were studied (Figure 6 and Additional file 2). Expression of Ccl2 (MCP-1), Ccl3 (MIP-1 α), Tlr (Toll-like receptor) 2 and Tlr4 as well as Tnf was up-regulated in ANKA*Δpm4-*infected placentas as compared to the other parasite lines. Conversely, placentas of K173- and NK65-infected females exhibited less reactive inflammatory response. This strongly suggests that ANKA*Δpm4* infection elicits an inflammatory response with a strong innate immunity component, which was not observed with parasites of the K173 and NK65 lines. Taken together, these results suggest that mechanisms involved in PM induction and progression might be different amongst different *P. berghei* lines and that these differences are not associated with the parasite burden in the placenta.

## Discussion

Mouse models amenable to genetic dissection of host factors of PM pathogenesis were established by analysing the severity of maternal infection, pregnancy outcome, placental pathology and the expression of inflammatory factors following infection with three *P. berghei* lines that do not induce cerebral malaria in the C57BL/6 mouse. The results provide evidence that infection of pregnant females with these *P. berghei* lines induces PM typical features, strongly suggesting that parasite factors determining cerebral malaria are not required to develop placental infection.

Nevertheless, the three parasite strains induced different degrees of placental pathology and impaired pregnancy outcome suggesting that parasite factors could underlie a spectrum of PM manifestations and distinct pathogenesis mechanisms. Thus, NK65 parasites induced the most severe syndrome comprising significantly lower foetal weight and decreased placental vascular area, higher percentage of nonviable foetuses per mother and lower number of live newborns. In addition, NK65 was the only parasite line causing maternal death before delivery (Table 1). Despite similar maternal parasitaemia in NK65 and K173-infected pregnant mice, the latter presented milder effects in placental pathology and in foetal weight loss. On the other hand, ANKA*Δpm4* infection led to lower stillbirth incidence and increased newborn viability compared to the other strains. These observations support the notion that different *P. berghei* lines show distinct patterns of PM. In fact, an heterogeneous and wide range of clinical manifestations is also observed in women that have malaria during pregnancy, including increased levels of parasitaemia [20-23], increased number of abortions, preterm delivery, intrauterine growth retardation, low birth weight, maternal mortality [24-28] and structural placenta alterations such as trophoblast thickening and consequent vascular space reduction [8,29]. Thus, the different *P. berghei* lines represent a fine-tuning resource in constructing experimental systems to study different aspects of pregnancy associated malaria pathogenesis.

It is widely accepted that accumulation of IE is a key event in the pathogenesis of severe disease as is the case of respiratory distress, CM and severe PM [1]. The experiments here presented confirmed that PM development was associated with parasite accumulation in the placenta. Nevertheless, the parasite burden in the placenta was not a major determinant of PM severity as the distinct pathology patterns observed in mice infected with NK65, K173 and ANKA*Δpm4* did not correlate with differences in placenta parasite accumulation. In particular, infection with the ANKA*Δpm4* line showed a lower impact on foetal viability despite a similar parasite burden in the placenta. An earlier report shows that, ANKA*Δpm4* parasites failed to induce disease in an ECM model but the resistance phenotype was correlated with lower parasite accumulation in the brain compared to wild-type *P. berghei* ANKA parasites. This virulence-attenuated effect was also observed in ECM-resistant mouse strains where self-resolving infection was associated to antibody-mediated response [16]. Nevertheless, this protective effect was not observed in ANKA*Δpm4-*infected pregnant mice although foetal viability was increased and correlated with a strong innate immune response. This raises the possibility that the vigorous local innate response in ANKA*Δpm4* infected placentas deterred the progression of placental tissue disorganization at least for a short period warranting an improved pregnancy outcome. Although expression of pro-inflammatory markers was less stimulated in K173- and NK65-infected placentas at G18 it is not ruled out the possibility that gene expression differences are not exclusively parasite line-related but could also be influenced by differences in parasite kinetics as parasite expansion in pregnant ANKA*Δpm4* mice was somewhat slower as compared to K173 and NK65. Thus, the observed differences in immune responses might also be influenced by the longer exposure of the maternal immune system to ANKA*Δpm4* (G10 to G19) as compared to K173 and NK65 parasite lines (G13 to G18). Nevertheless, the PM protracting effects observed in ANKA*Δpm4* infection offer now interesting research perspectives. This experimental model can be used to (1) discriminate between the effects exerted by foetal- and maternal-derived inflammatory factors in PM pathogenesis and (2) to ascertain whether innate immune responses can be used to provide effective foetal protection in PM.

## Conclusions

The experiments here presented made used of three different *P. berghei* lines and show that parasite components that induce pathology during pregnancy are distinct from those that induce experimental cerebral malaria. In addition, the data indicate that PM pathology in ANKA*Δpm4* infected mice is associated with an inflammatory response with strong innate immune component, which was not observed in K173- and NK65-infected pregnant mice. The characterization of different experimental systems of PM in the C57BL/6 mouse will allow interrogation of genetically modified mice to ascertain the role of host molecules in PM pathogenesis and to dissect foetal and maternal contributions in placental pathology.

## Abbreviations

PM: placental malaria; CM: cerebral malaria; IE: infected erythrocytes; ECM: experimental cerebral malaria.

## Competing interests

All authors have declared no conflict of interest.

## Authors’ contributions

LRD and LVM conceived and designed the research/study, performed experiments, analysed data and wrote the paper; CPG conceived and designed the research/study, analysed data, wrote the paper; CRFM and RB performed morphometric analysis, analysed and discussed data; BFF and CJJ provided materials, reviewed and discussed the experimental data and wrote the paper. All authors read and approved the manuscript.

## Supplementary Material

Additional file 1 **Table.** Infection during pregnancy increases the percentage of stillbirths. Click here for file

Additional file 2 **Figure.** Gene expression of inflammatory factors in non-infected placentas. Placentas from healthy pregnant females were collected at G18 or G19 and RNA expression of Ccl2, Ccl3, Tlr2, Tlr4 and Tnfα genes were evaluated by qReal Time PCR. Relative quantification (RQ) was obtained with normalization by GAPDH. *p<0.05. Click here for file
