# Supplementary material for: Distinct placental malaria pathology caused by different Plasmodium berghei lines that fail to induce cerebral malaria in the C57BL/6 mouse
Source: Malar J. 2012 Jul 16;11:231. doi: 10.1186/1475-2875-11-231 (PMC3485172; doi:10.1186/1475-2875-11-231)
Supplement: Additional file 1 — Table. Infection during pregnancy increases the percentage of stillbirths. [file 1475-2875-11-231-S1.doc]

**Additional file 2.** Infection during pregnancy increases the percentage of stillbirths

|  | **Number of mothers** | **Number of stillbirths** | **% stillbirths/mother** |
| --- | --- | --- | --- |
| **Non-infected** | 6 | 4 | 0,67 |
| **K173** | 13 | 64 | 4,92 |
| **NK65** | 16 | 91 | 5,69 |
| **ANKA*∆pm4*** | 8 | 14 | 1,75 |
