# Supplementary figures and images for: Distinct placental malaria pathology caused by different Plasmodium berghei lines that fail to induce cerebral malaria in the C57BL/6 mouse
Source: Malar J. 2012 Jul 16;11:231. doi: 10.1186/1475-2875-11-231 (PMC3485172; doi:10.1186/1475-2875-11-231)

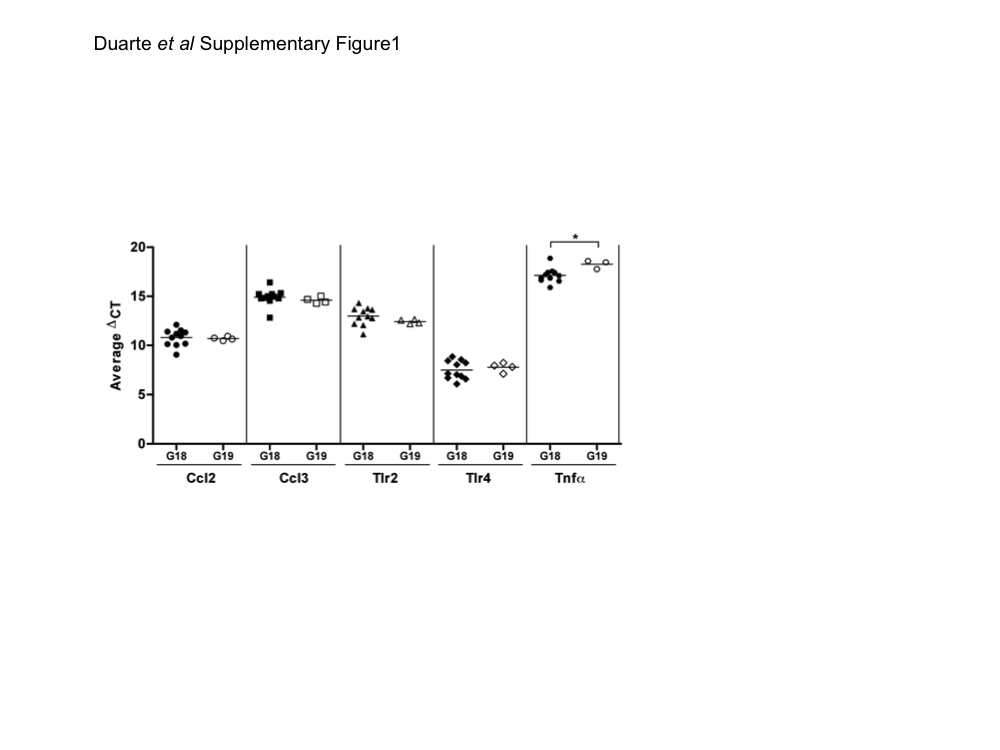

Supplement: Additional file 2 — Figure. Gene expression of inflammatory factors in non-infected placentas. Placentas from healthy pregnant females were collected at G18 or G19 and RNA expression of Ccl2, Ccl3, Tlr2, Tlr4 and Tnfα genes were evaluated by qReal Time PCR. Relative quantification (RQ) was obtained with normalization by GAPDH. *p<0.05. [file 1475-2875-11-231-S2.tiff]
